# Supplementary material for: Systems genetics in diversity outbred mice inform BMD GWAS and identify determinants of bone strength
Source: Nat Commun. 2021 Jun 7;12:3408. doi: 10.1038/s41467-021-23649-0 (PMC8184749; doi:10.1038/s41467-021-23649-0)

Supplemental Figure 1. Principal Component Analysis of bulk RNA-seq data. a) Scree plot showing the percentage of explained variance for the first 10 principal components. **b**) Individuals in PC1 and PC2 space, colored by sex. **c**) Individuals in PC3 and PC4 space, colored by sex. **d**) Individuals in PC1 and PC2 space, colored by batch. **e**) Individuals in PC1 and PC2 space, colored by batch. **f**) Individuals in PC1 and PC2 space, colored by age (binarized, see Methods). **g**) Individuals in PC3 and PC4 space, colored by age (binarized, see Methods).

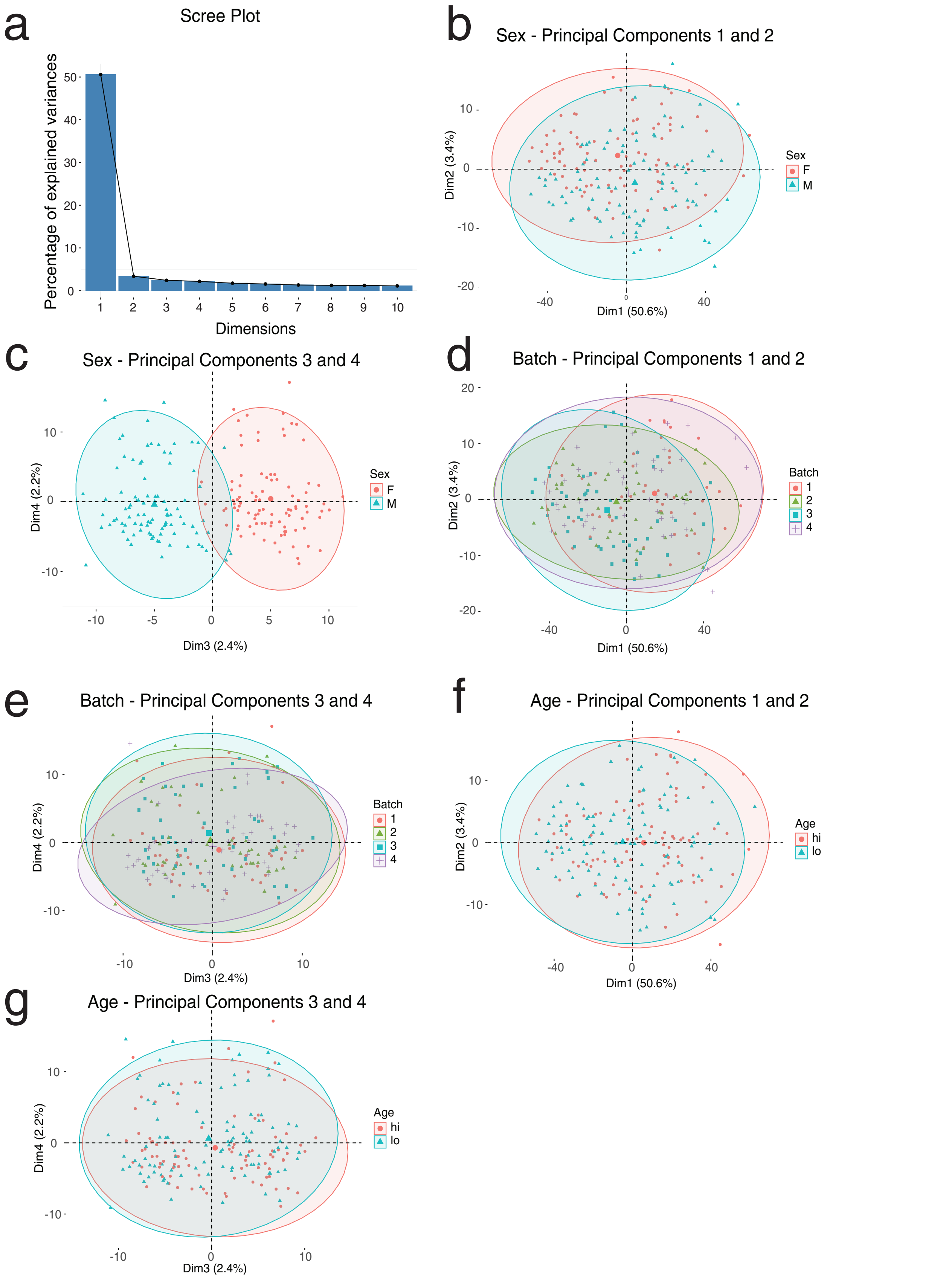

Supplemental Figure 2. Mineralization of bone marrow-derived stromal cells exposed to osteogenic differentiation media *in vitro*. During differentiation, cells from each individual DO mouse were assessed for accumulated mineralization by IRDye 680 BoneTag Optical Probe incorporation. The final values for mineralization shown here were computed by subtracting the average number of fluorescent units recorded in designated background wells from the number of fluorescent units recorded in the sample wells. In the cultures from DO mouse #50, there was a much higher percentage of marrow adipogenic lineage precursor cells and a small number of osteoblasts. Consistent with this observation, mouse #50 also demonstrated high levels of marrow adiposity. This is likely the basis of the poor in vitro mineralization observed for the cultures from this mouse.

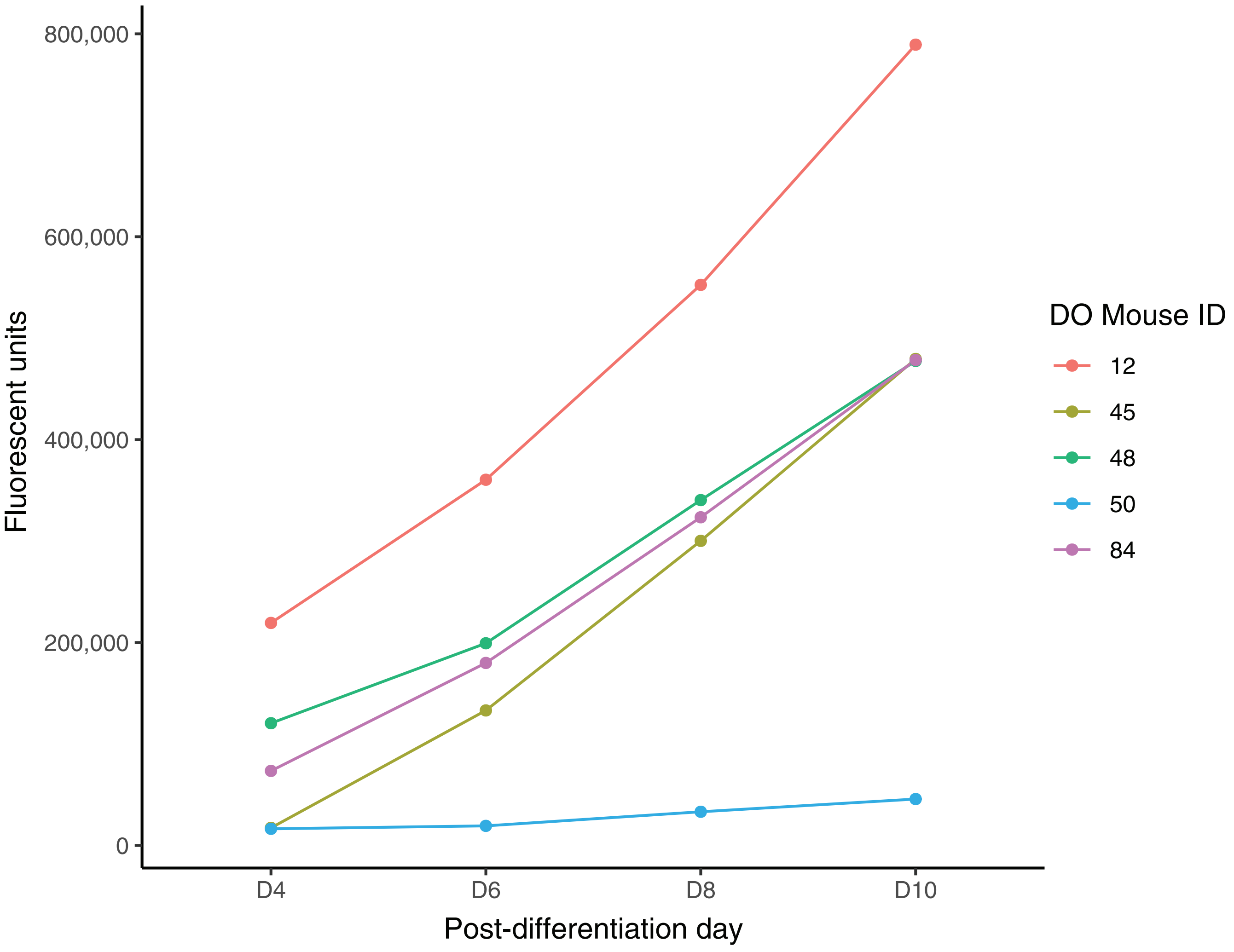

Supplemental Figure 3. Sex-specific UMAP visulization of single cell RNA-seq expression data on bone marrow stromal cellls cultured in osteogenic differentiation media *in vitro.* Each point represents a cell. **a**) *Sertad4* expression in cells from a male DO mouse. **b**) *Glt8d2* expression in cells from female DO mice (N=4). **c**) *Sertad4* expression in cells from a male DO mouse. **d**) *Glt8d2* expression in cells from female DO mice (N=4). In all panels, the color scales indicate normalized gene expression values.

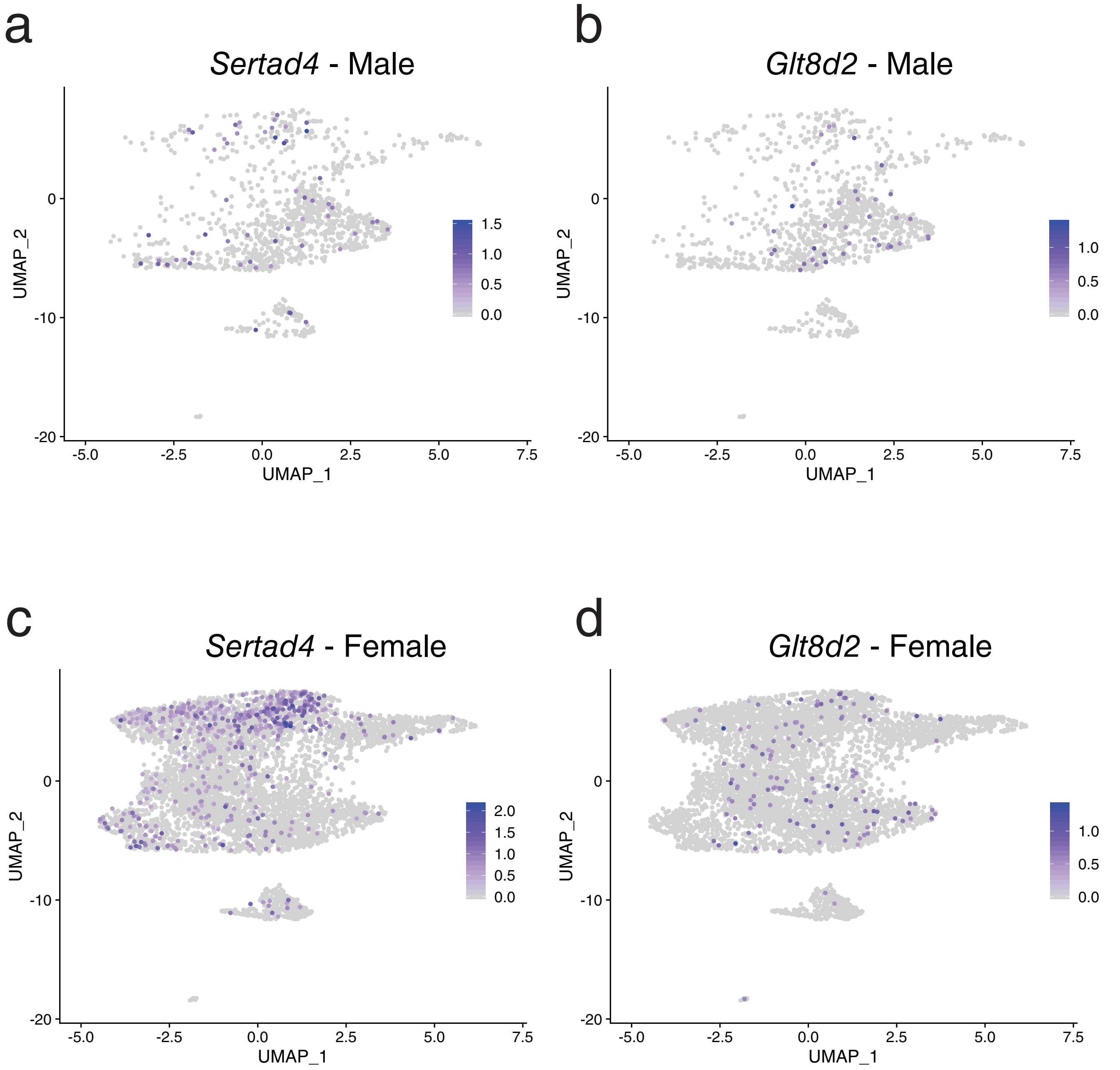

Supplemental Figure 4. Significant QTL associations. Twenty-eight mapped QTL exceeding permutation-based LOD score thresholds (alpha=0.05)

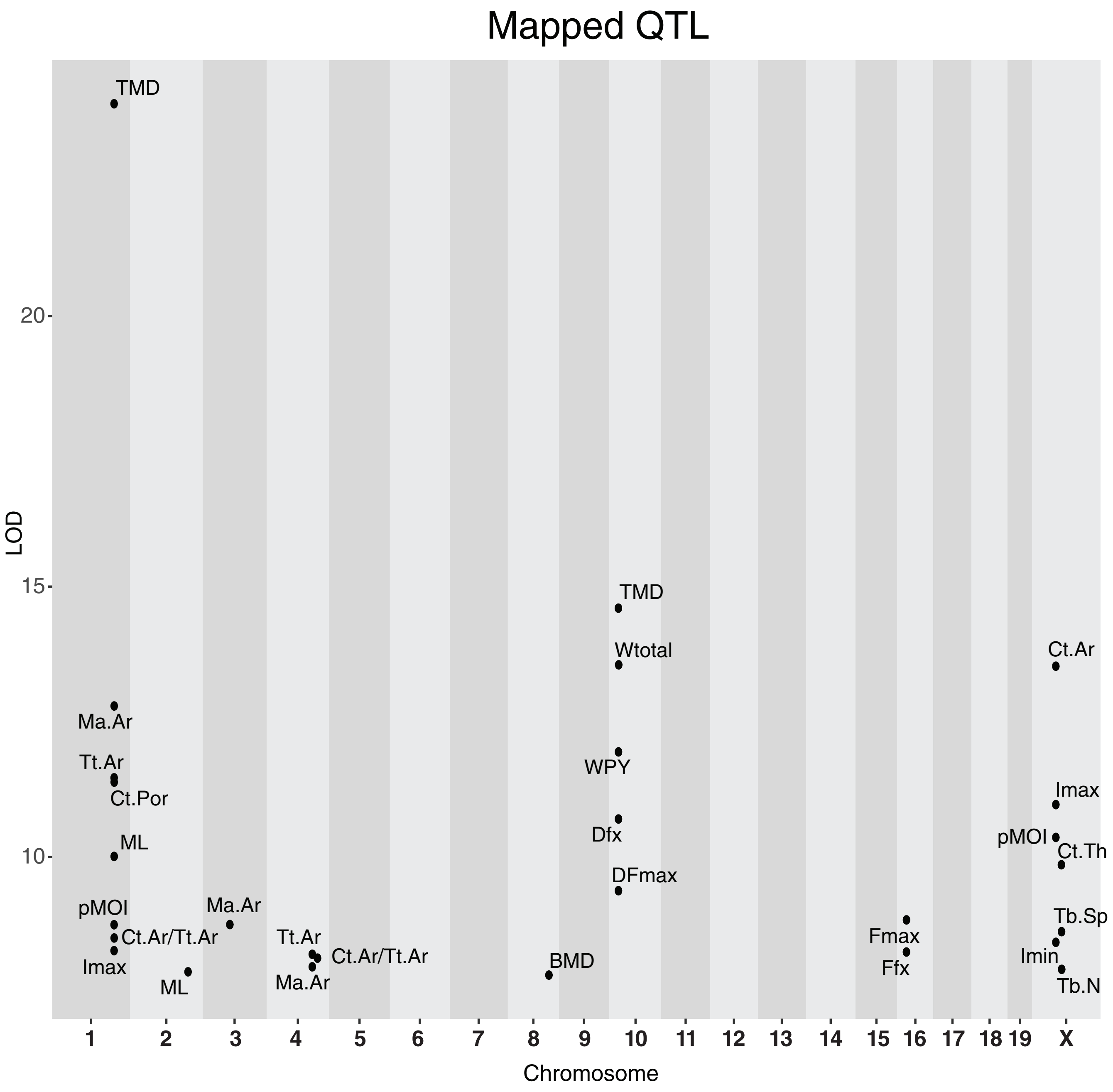

Supplemental Figure 5. Overlap between BMD GWAS SNPs and QTL loci. Each panel corresponds to a QTL locus's syntenic human region. Panels a-j represent each of the 10 loci sequentially. Red circles represent BMD GWAS SNPs in the locus. The horizontal lines represent the genome-wide significance threshold ($P = 5x10^{-8}$). Not all genes are shown.

a

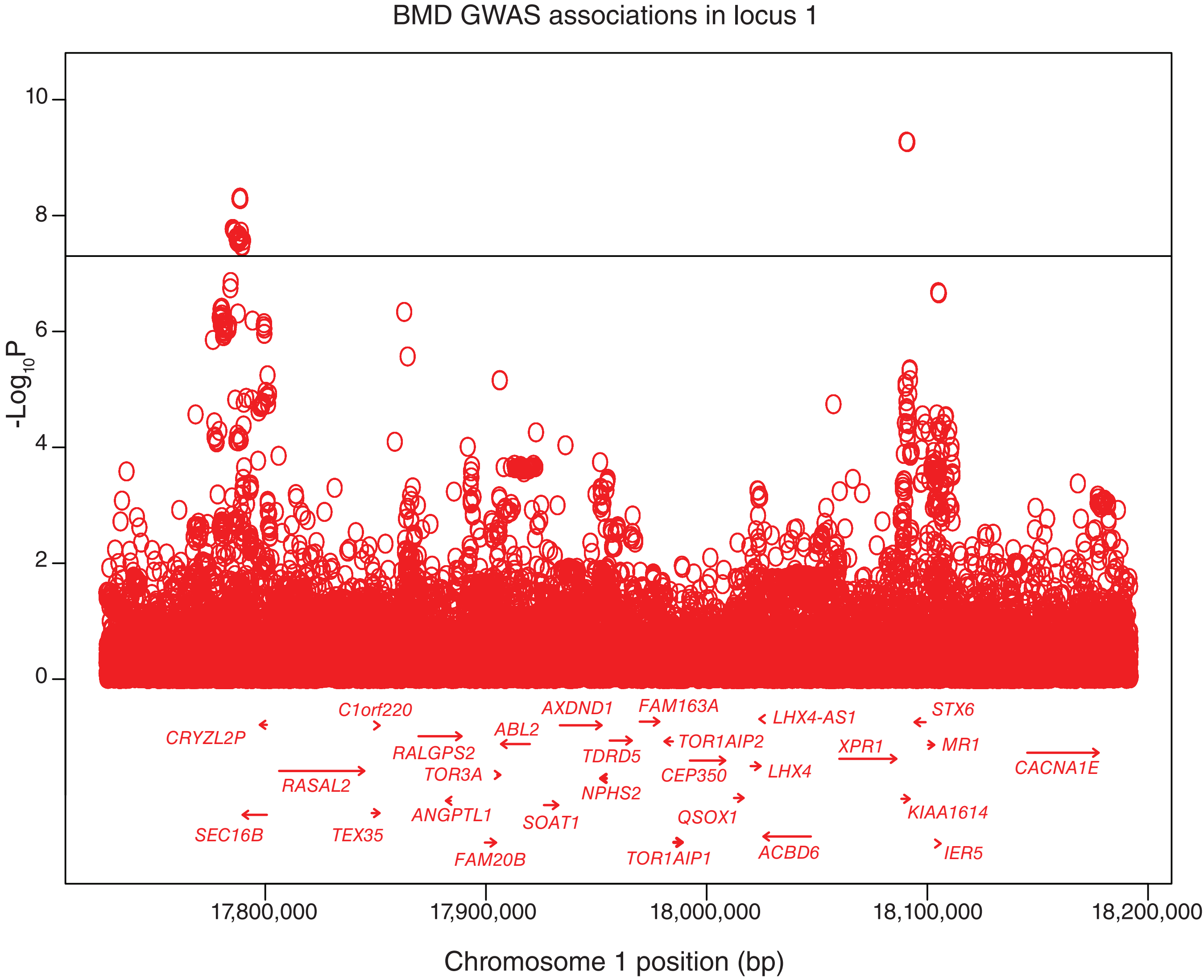

b

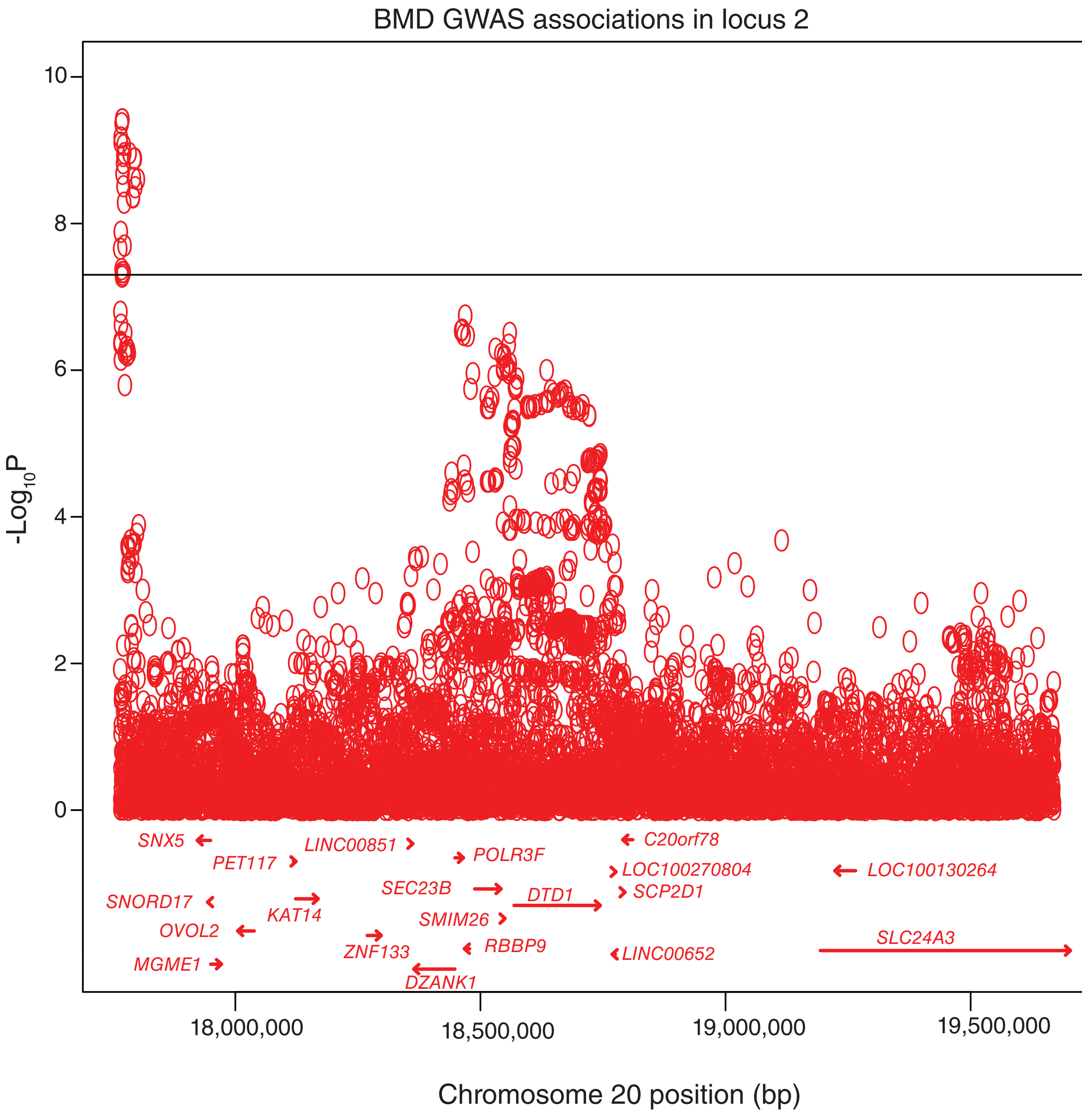

BMD GWAS associations in locus 2
10
8
6
4
2
0
-Log10P
SNX5
PET117
LINC00851
POLR3F
C20orf78
LOC100270804
LOC100130264
SNORD17
SEC23B
DTD1
SCP2D1
KAT14
SMIM26
OVOL2
SLC24A3
ZNF133
RBBP9
LINC00652
MGME1
DZANK1
18,000,000
18,500,000
19,000,000
19,500,000
Chromosome 20 position (bp)

c

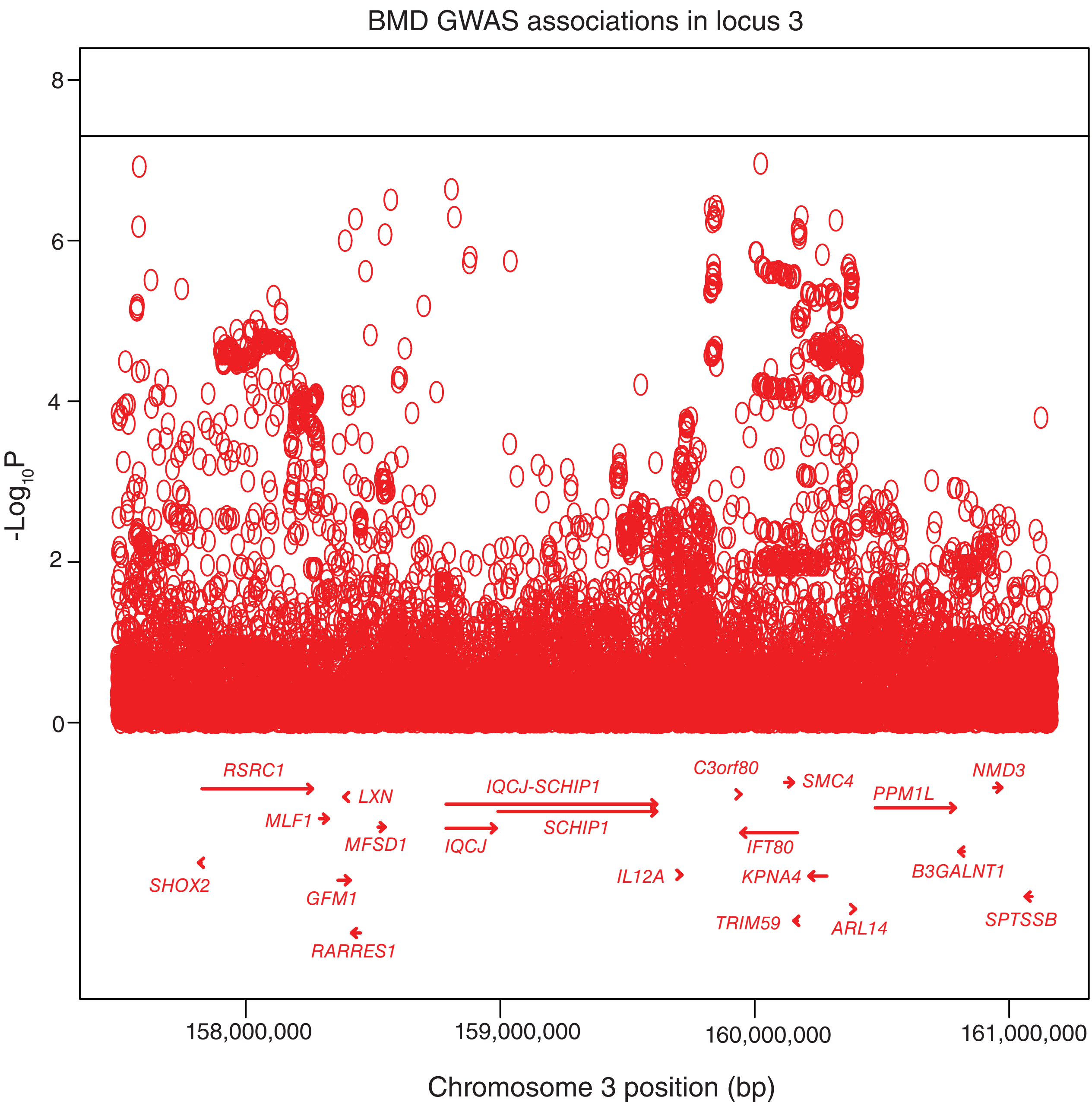
BMD GWAS associations in locus 3
-Log10P
8
6
4
2
0
RSRC1
LXN
MLF1
MFSD1
SHOX2
GFM1
RARRES1
IQCJ-SCHIP1
SCHIP1
IQCJ
IL12A
C3orf80
SMC4
PPM1L
NMD3
IFT80
KPNA4
TRIM59
ARL14
B3GALNT1
SPTSSB
158,000,000
159,000,000
160,000,000
161,000,000
Chromosome 3 position (bp)

d

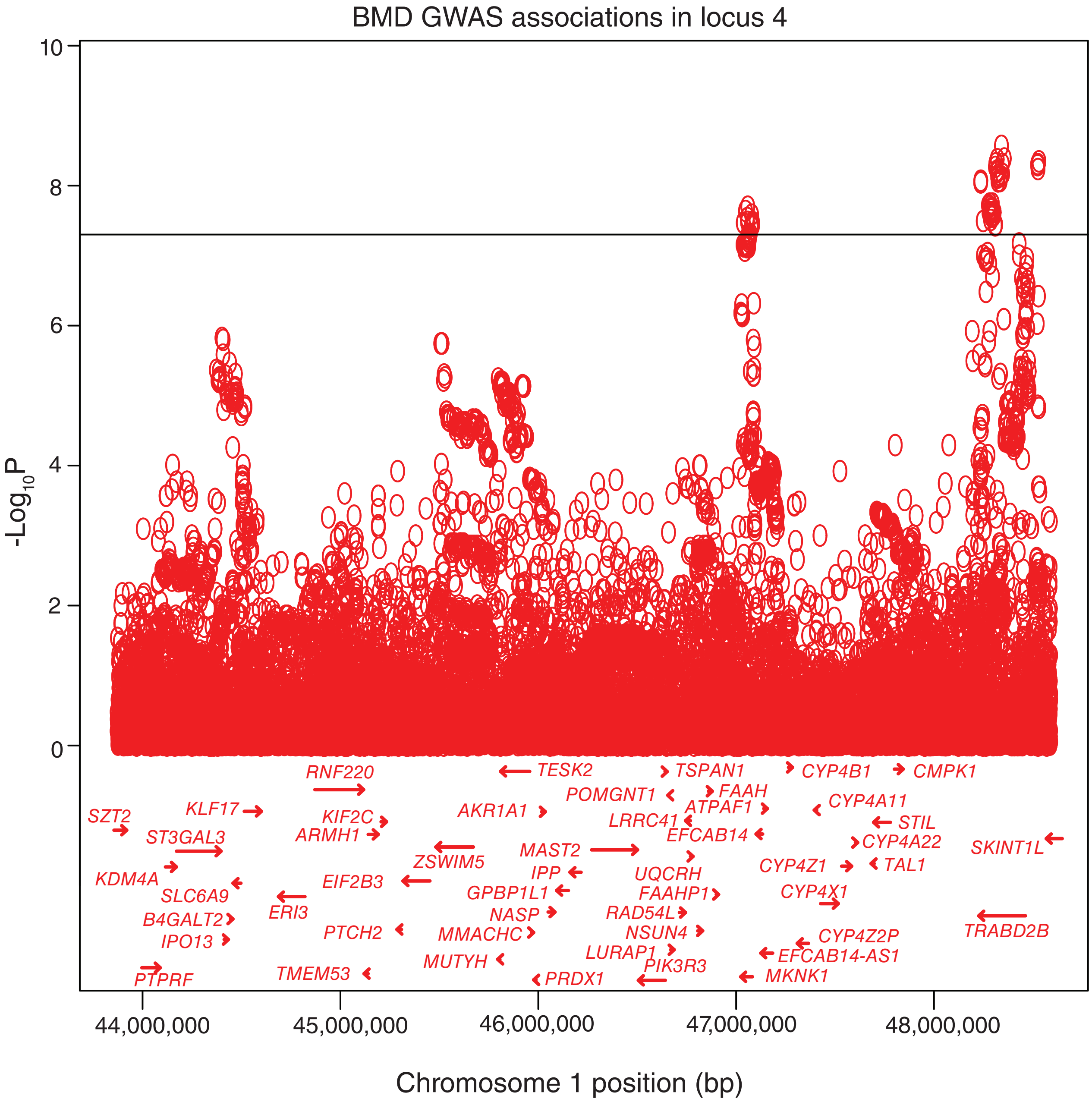
BMD GWAS associations in locus 4
-Log10P
0
2
4
6
8
10
RNF220
TESK2
TSPAN1
CYP4B1
CMPK1
POMGNT1
FAAH
CYP4A11
SZT2
KLF17
KIF2C
AKR1A1
ATPAF1
STIL
LRRC41
ST3GAL3
ARMH1
EFCAB14
CYP4A22
SKINT1L
MAST2
ZSWIM5
CYP4Z1
TAL1
KDM4A
IPP
UQCRH
EIF2B3
SLC6A9
GPBP1L1
FAAHP1
CYP4X1
ERI3
NASP
RAD54L
B4GALT2
TRABD2B
PTCH2
MMACHC
NSUN4
CYP4Z2P
IPO13
LURAP1
EFCAB14-AS1
MUTYH
PIK3R3
TMEM53
MKNK1
PTPRF
PRDX1
44,000,000
45,000,000
46,000,000
47,000,000
48,000,000
Chromosome 1 position (bp)

e

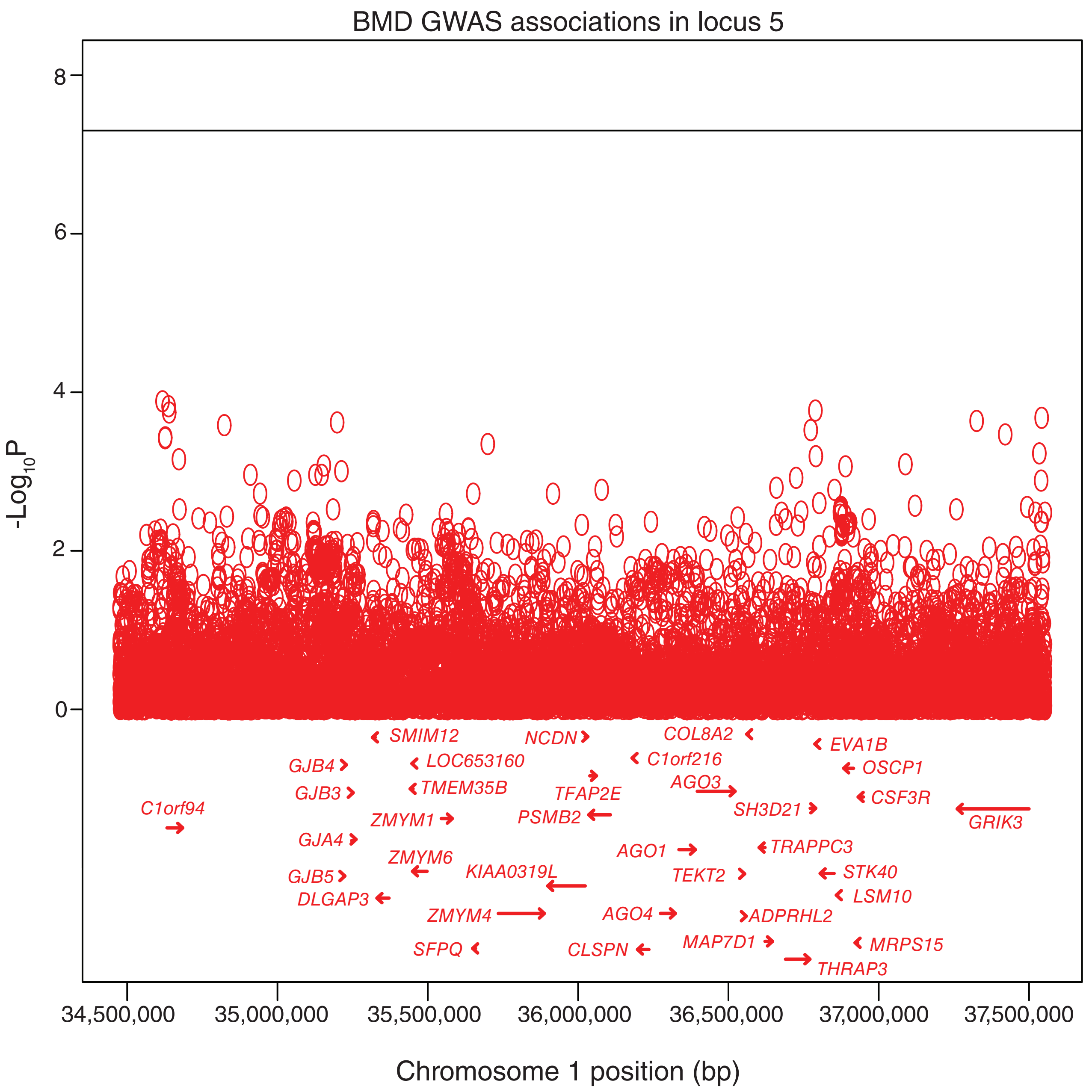

BMD GWAS associations in locus 5
-Log10P
8
6
4
2
0
SMIM12
NCDN
COL8A2
EVA1B
GJB4
LOC653160
C1orf216
OSCP1
GJB3
TMEM35B
TFAP2E
AGO3
CSF3R
C1orf94
ZMYM1
PSMB2
SH3D21
GRIK3
GJA4
AGO1
TRAPPC3
ZMYM6
KIAA0319L
TEKT2
STK40
GJB5
DLGAP3
LSM10
ZMYM4
AGO4
ADPRHL2
SFPQ
CLSPN
MAP7D1
MRPS15
THRAP3
34,500,000
35,000,000
35,500,000
36,000,000
36,500,000
37,000,000
37,500,000
Chromosome 1 position (bp)

f

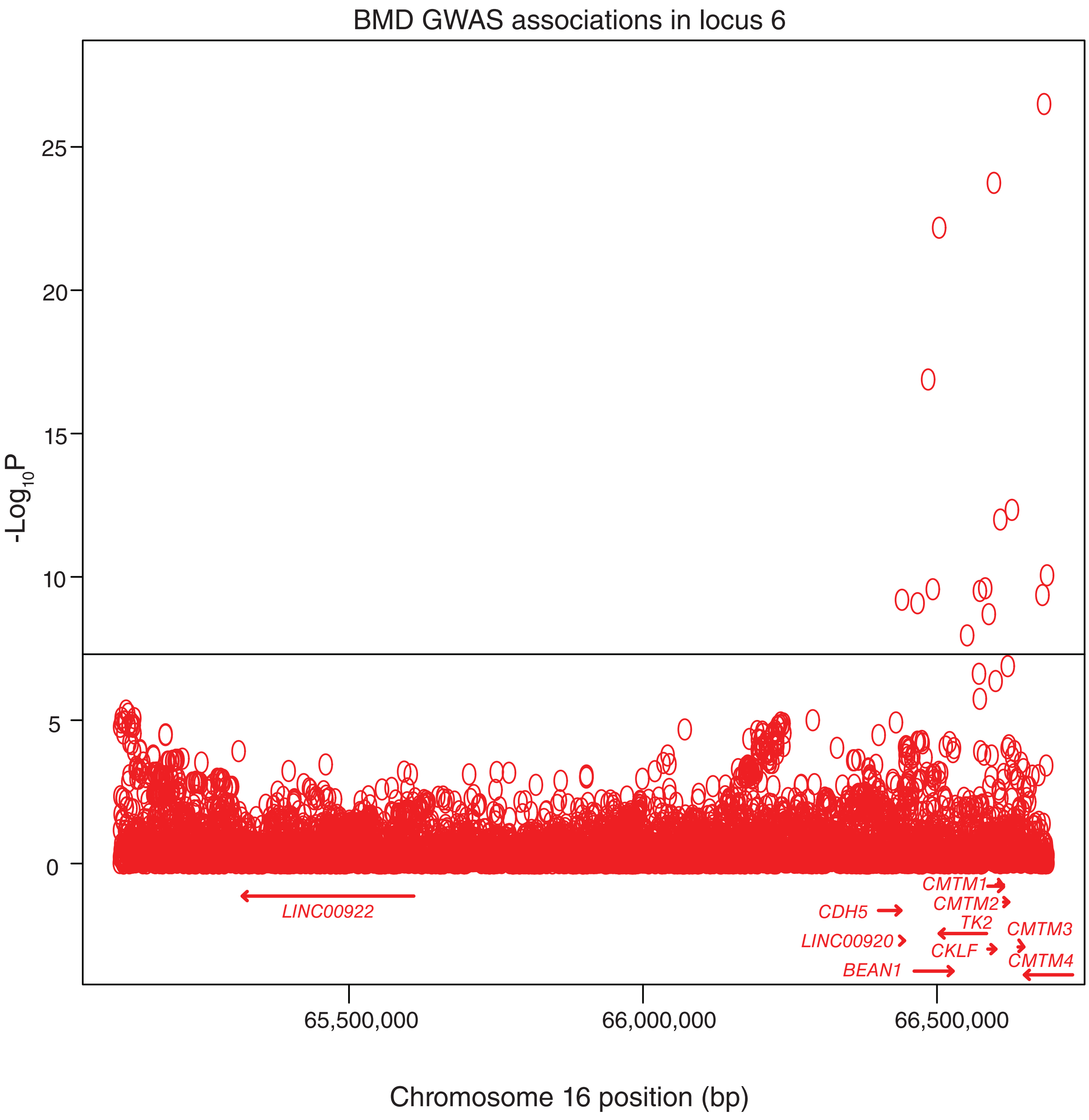
BMD GWAS associations in locus 6
25
20
15
10
5
0
-Log10P
LINC00922
CMTM1
CDH5
CMTM2
TK2
CMTM3
LINC00920
CKLF
CMTM4
BEAN1
65,500,000
66,000,000
66,500,000
Chromosome 16 position (bp)

g

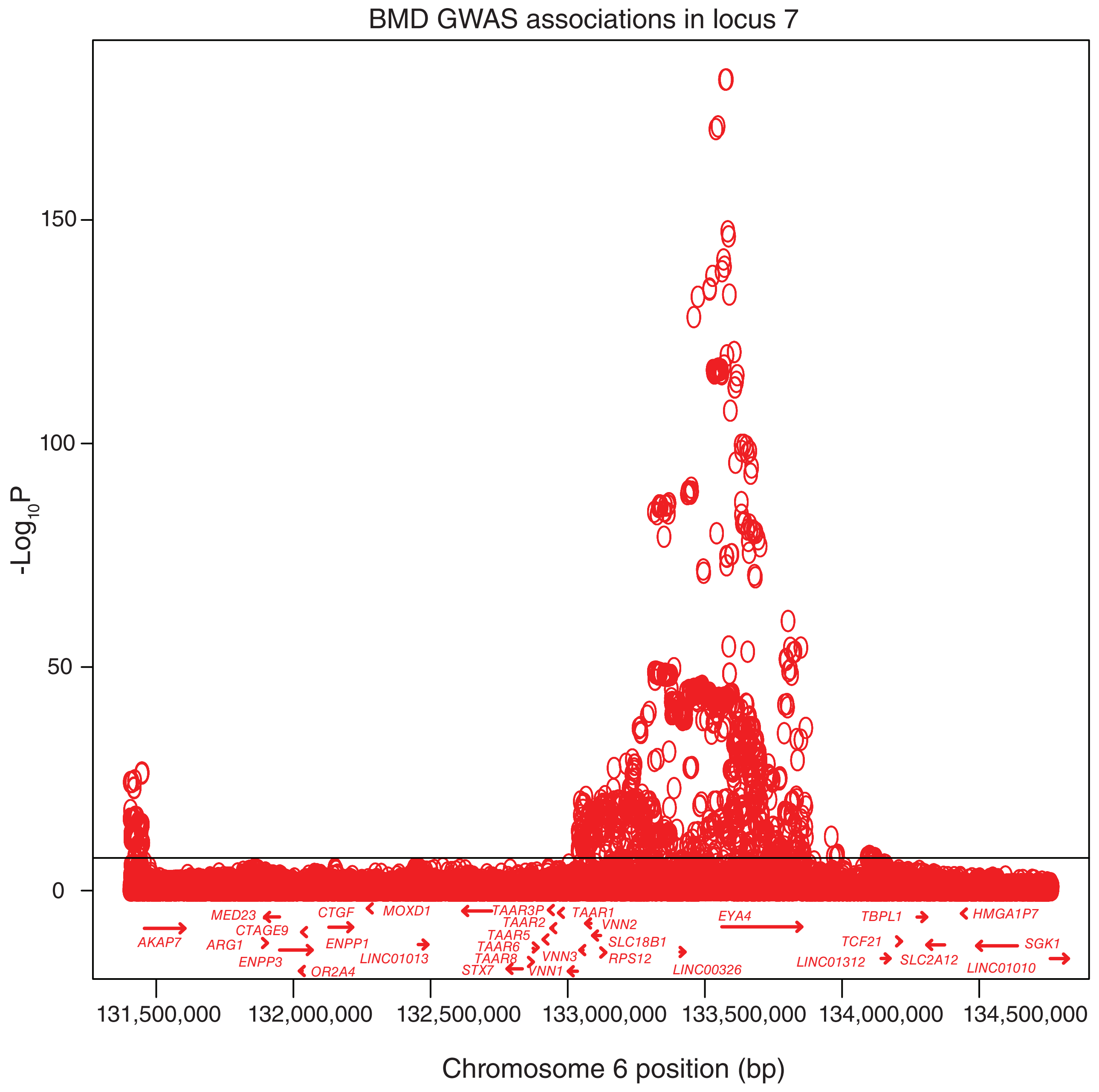
BMD GWAS associations in locus 7
-Log10P
150
100
50
0
AKAP7
MED23
CTAGE9
ARG1
ENPP3
OR2A4
CTGF
ENPP1
MOXD1
LINC01013
TAAR3P
TAAR2
TAAR5
TAAR6
TAAR8
STX7
TAAR1
VNN2
SLC18B1
VNN3
VNN1
RPS12
LINC00326
EYA4
TBPL1
TCF21
LINC01312
SLC2A12
HMGA1P7
SGK1
LINC01010
131,500,000
132,000,000
132,500,000
133,000,000
133,500,000
134,000,000
134,500,000
Chromosome 6 position (bp)

h

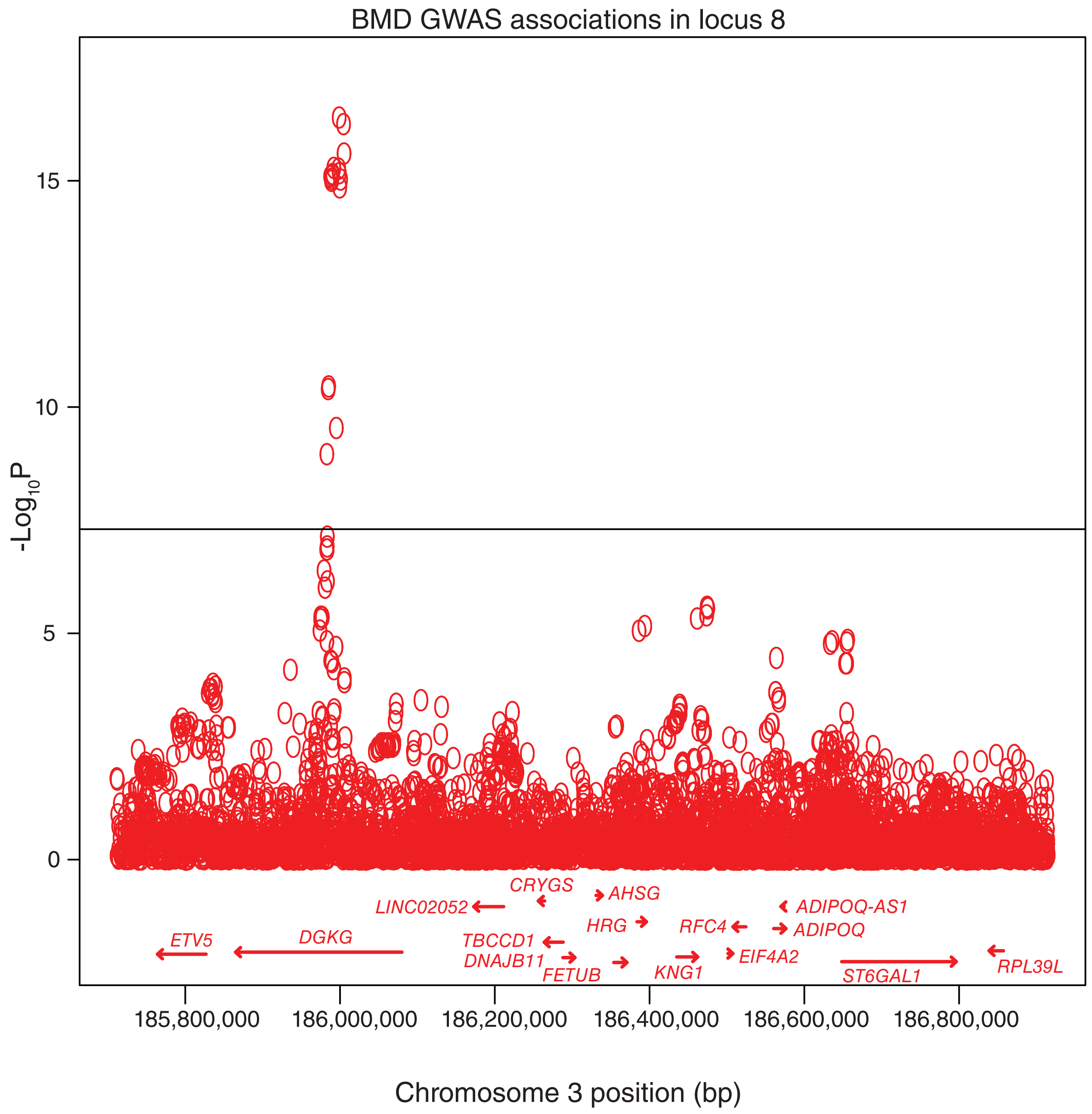
BMD GWAS associations in locus 8
-Log10P
15
10
5
0
CRYGS
AHSG
LINC02052
ADIPOQ-AS1
HRG
RFC4
ADIPOQ
ETV5
DGKG
TBCCD1
DNAJB11
EIF4A2
FETUB
KNG1
ST6GAL1
RPL39L
185,800,000
186,000,000
186,200,000
186,400,000
186,600,000
186,800,000
Chromosome 3 position (bp)

i

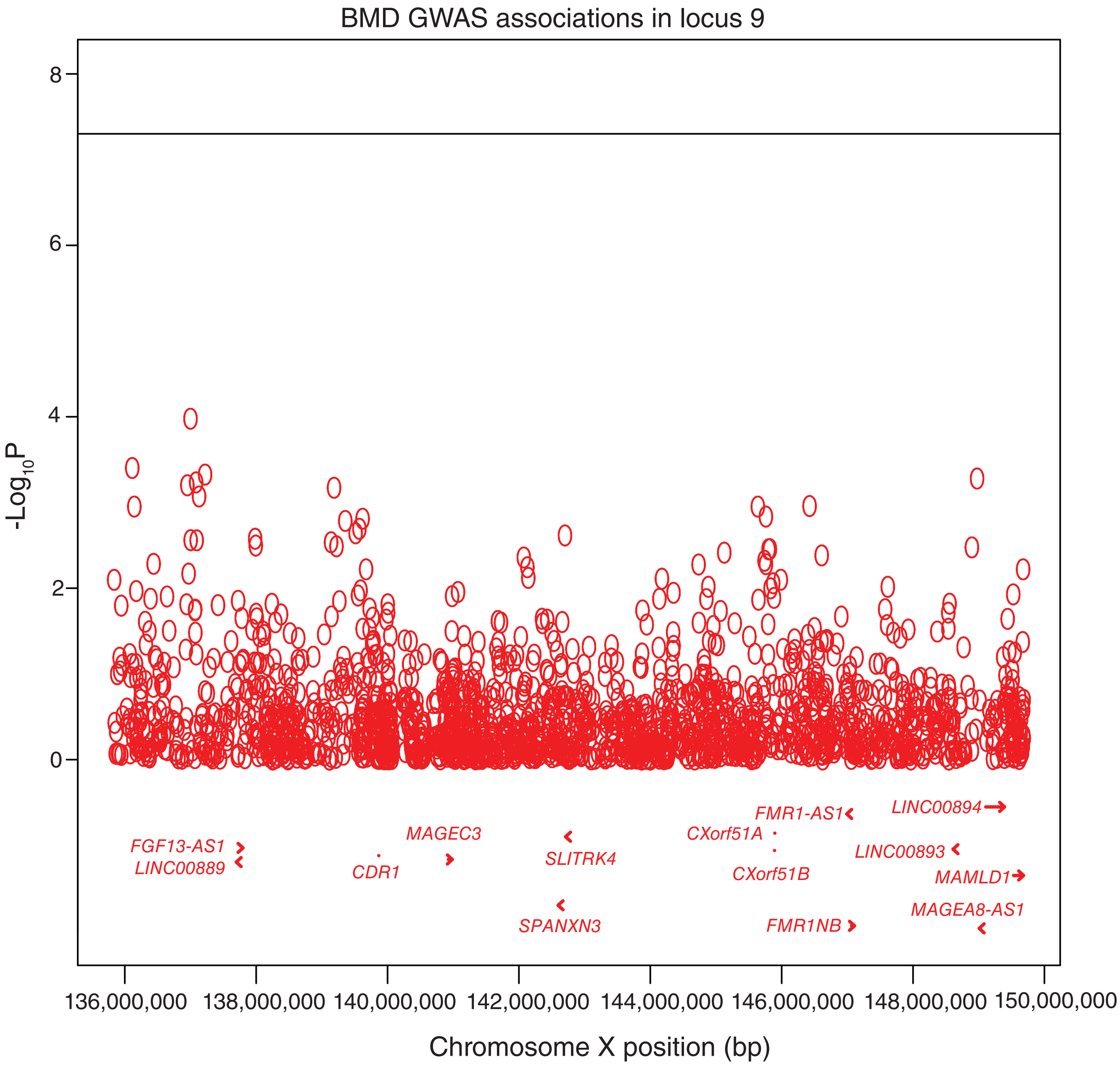
BMD GWAS associations in locus 9
-Log10P
8
6
4
2
0
FGF13-AS1
LINC00889
CDR1
MAGEC3
SLITRK4
SPANXN3
CXorf51A
CXorf51B
FMR1-AS1
FMR1NB
LINC00894
LINC00893
MAMLD1
MAGEA8-AS1
136,000,000
138,000,000
140,000,000
142,000,000
144,000,000
146,000,000
148,000,000
150,000,000
Chromosome X position (bp)

j

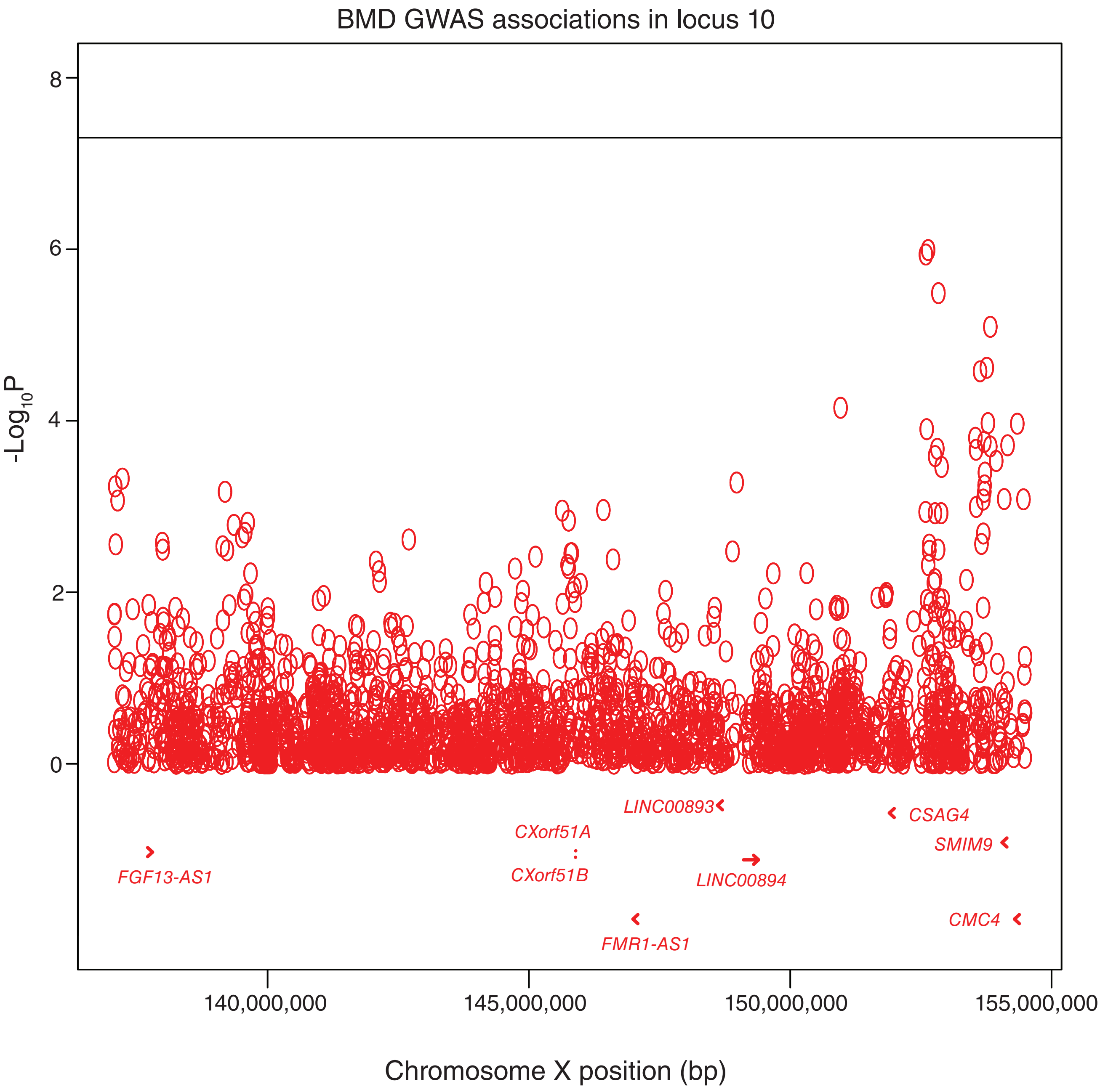

BMD GWAS associations in locus 10
-Log10P
8
6
4
2
0
LINC00893
CSAG4
CXorf51A
SMIM9
FGF13-AS1
CXorf51B
LINC00894
CMC4
FMR1-AS1
140,000,000
145,000,000
150,000,000
155,000,000
Chromosome X position (bp)

Supplemental Figure 6. ML mapping in a replication cohort. The top panel shows allele effects for the DO founders for ML in an interval on chromosome 1 (Mbp). Y-axis units are best linear unbiased predictors (BLUPs). The bottom panel shows the QTL scan.

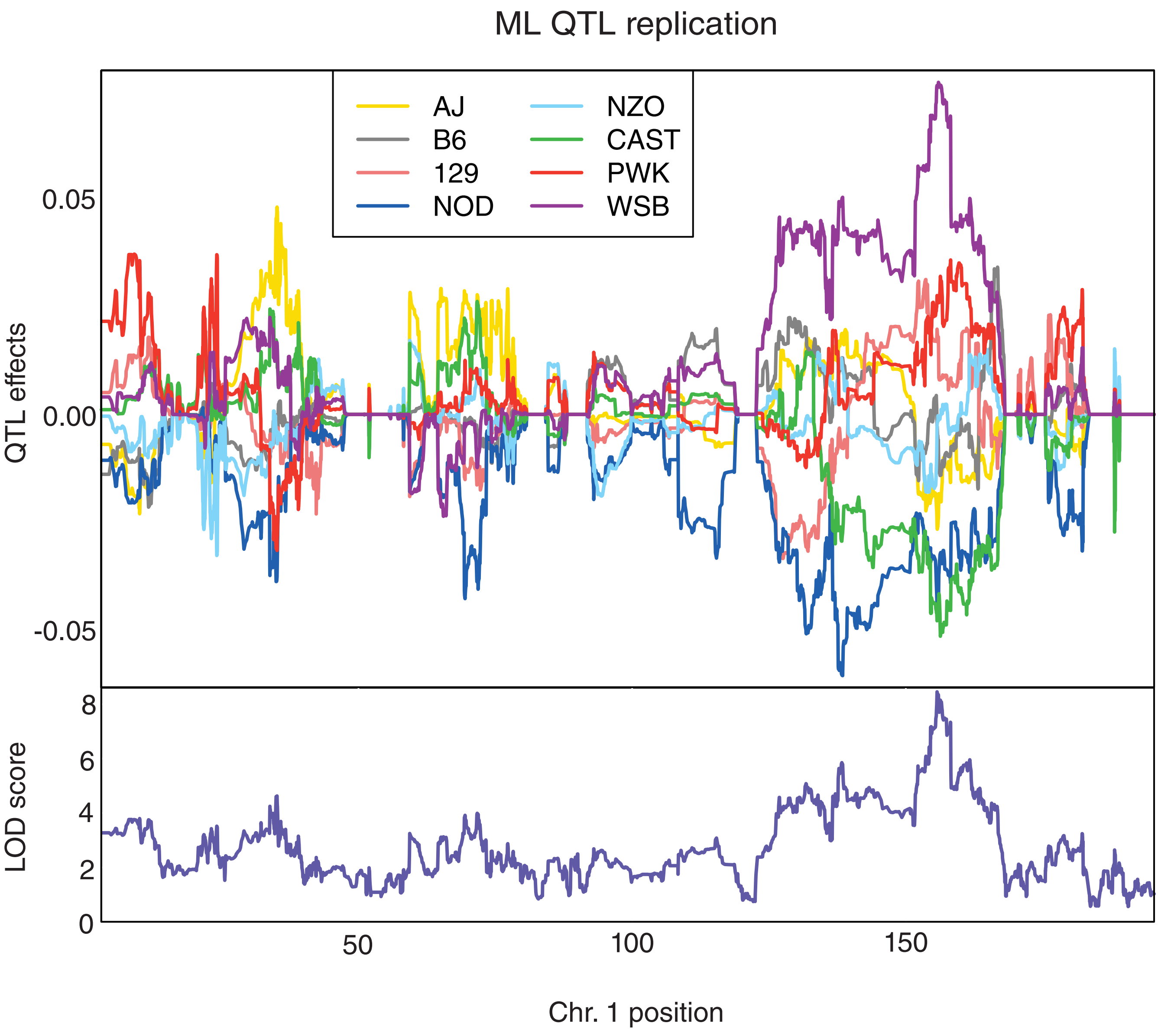

Supplement: Supplementary file 1 — Supplementary Information [file 41467_2021_23649_MOESM1_ESM.pdf]
