# Supplementary material for: Systems genetics in diversity outbred mice inform BMD GWAS and identify determinants of bone strength
Source: Nat Commun. 2021 Jun 7;12:3408. doi: 10.1038/s41467-021-23649-0 (PMC8184749; doi:10.1038/s41467-021-23649-0)
Supplement: Supplementary file 3 — Description of Additional Supplementary Files [file 41467_2021_23649_MOESM3_ESM.pdf]

Description of Additional Supplementary Files

File Name: Supplementary Data 1
Description: Phenotypes collected in the DO. Includes phenotype, abbreviation, measurement units, number measured and number mapped. “Number measured” corresponds to the number of measurements available per phenotype, while “number mapped” indicates the number of measurements included in the QTL mapping. Since QTL mapping utilizes covariates, mice with missing covariates (such as weight) were excluded from mapping.

File Name: Supplementary Data 2
Description: Raw measurements of the 55 mapped phenotypes, as well as other phenotypes such as weight and age at sacrifice.

File Name: Supplementary Data 3
Description: Correlations of mapped phenotypes with bone strength (max load, Fmax). Spearman correlations, Holm-Bonferroni adjusted (two-sided) p-values, and standard error are reported. Some phenotypes are transformed (see Methods).

File Name: Supplementary Data 4
Description: Pairwise correlations of all 55 mapped phenotypes. Spearman correlations are reported, with Holm-Bonferroni adjusted (two-sided) p-values in parentheses. Some phenotypes are transformed (see methods).

File Name: Supplementary Data 5
Description: Results of bulk RNA-seq differential expression between sexes (M vs F). Genes with an adjusted P-value <= 0.05 are reported. Columns are gene name, MGI ID, ENSEMBL ID for genes with missing MGI IDs, the average of the normalized count values dividing by size factors taken over all samples, the shrunken log2 fold change between groups, the standard error of the log2 fold change, p-value, and the adjusted p-value (Benjamini-Hochberg).

File Name: Supplementary Data 6
Description: Results of bulk RNA-seq differential expression between individuals with low vs high bone strength (see methods). Genes with an adjusted P-value <= 0.05 are reported. Columns are gene name, MGI ID, the average of the normalized count values dividing by size factors taken over all samples, the shrunken log2 fold change between groups, the standard error of the log2 fold change, p-value, and the adjusted p-value (Benjamini-Hochberg).

File Name: Supplementary Data 7
Description: Module membership. Pearson correlations between module eigengenes and gene expression are reported for all genes across 124 modules. Suffixes describe sex-combined, female or male networks (“_C”, “_F” and “_M”, respectively). In a few cases, there is no MGI accession ID, so an Ensembl ID is provided instead.

File Name: Supplementary Data 8
Description: Modular Gene Ontology terms with a p-value <= 0.05. GO terms and p-values are reported. P-values were calculated using a one-sided Fisher's exact test, and were not adjusted for multiple comparisons. Column names are WGCNA module identifiers. Suffixes describe the network from in

which a particular module is found (Sex-combined, female or male networks , "_C", "_F" and "_M", respectively).

File Name: Supplementary Data 9
Description: List of known bone genes.

File Name: Supplementary Data 10
Description: Bayesian networks. For each gene in our dataset, the Bayesian networks of which the gene is a member are reported. Also reported are the number of neighbors, number of neighbors that are bone genes, nominal (unadjusted) and multiple comparison-adjusted (FDR) p-values, and whether the gene was one of the 1,370 BANs. P-values were calculated using the hypergeometric distribution (one-sided). Suffixes describe sex-combined, female or male networks ("_C", "_F" and "_M", respectively). In a few cases, there is no MGI accession ID, so an Ensembl ID is provided instead.

File Name: Supplementary Data 11
Description: Significantly colocalizing genes. Genes with coloc H4 posterior probabilities >= 0.75 are reported, along with the GWAS phenotype with which they colocalized, the GTEx tissue in which they colocalized, and all coloc posterior probabilities.

File Name: Supplementary Data 12
Description: Homologous human BANs with colocalizing eQTL. Columns are gene name (genes in bold are known bone genes), Ensembl ID, WGCNA module with the most significant BAN p-value, the H4 probability from coloc, the number of neighbors in the Bayesian network, the number of known bone neighbors in the Bayesian network, the BAN P-value, which was calculated using a (one-sided) hypergeometric distribution and was not adjusted for multiple comparisons, and the tissue with the highest coloc H4 probability.

File Name: Supplementary Data 13
Description: Spearman correlations between WGCNA modules and phenotypes (raw values). P-values are presented in parentheses, and are calculated using the Student's t-test (two-sided). P-values were not adjusted for multiple comparisons.

File Name: Supplementary Data 14
Description: Mineralization readouts (fluorescent units) for bone marrow stromal cells exposed to osteogenic differentiation media in vitro. Values are computed by subtracting the average number of fluorescent units recorded in designated background wells from the number of fluorescent units in the sample wells. Columns are sample ID (DO mouse ID) and mineralization values for days 4, 6, 8, and 10.

File Name: Supplementary Data 15
Description: Marker genes for each of the clusters in the scRNA-seq data, as defined by differential expression. Positively differentially expressed genes are reported. Columns are gene name, MGI ID, ENSEMBL ID for genes without an MGI ID, unadjusted pvalue calculated using a two-sided Wilcoxon Rank Sum test, log fold change of the average expression between the relevant cluster and all other clusters, percentage of cells in the relevant cluster where the gene is detected, percentage of cells where the gene is detected in all other clusters, multiple-comparison adjusted p-value based on Bonferroni correction using all genes in the dataset, and the relevant cluster ID.

File Name: Supplementary Data 16

Description: Syntenic DO loci. The 10 DO QTL loci are reported (GRCm38/mm10), as well as their syntenic human counterparts (GRCh37/hg19), as obtained by the UCSC liftOver tool.

File Name: Supplementary Data 17
Description: Variant effect predictor (VEP) output for 15 missense SNPs.

File Name: Supplementary Data 18
Description: Mapped eQTL. Mapped local eQTL peaks in DO mice are reported, including eQTL position, LOD score, LOD support intervals, and distance from gene start coordinates (GRCm38).

File Name: Supplementary Data 19
Description: ScRNA-seq cluster 1 genes. Positively differentially expressed genes within the Qsox1 scRNA-seq cluster are reported. Columns are gene and MGI ID, unadjusted p-value calculated using a two-sided Wilcoxon Rank Sum test, log fold change of the average expression between cluster 1 and all other clusters, percentage of cells in cluster 1 where the gene is detected, percentage of cells where the gene is detected in all other clusters, and the p-value after adjustment for multiple comparisons (Bonferroni).

File Name: Supplementary Data 20
Description: Founder mice for CRISPR/Cas9 analysis.

File Name: Supplementary Data 21
Description: Predicted amino acid sequences for Qsox1 deletion mutants. Signal peptides are underlined in the wild type sequence, and catalytic residues are in bold, in the wild type sequence. The amino acid position column is the position of the last amino acid in the preceding sequence.

File Name: Supplementary Data 22
Description: Oligonucleotide sequences for CRISPR/Cas9 analysis.
