# Supplementary material for: Systems genetics in diversity outbred mice inform BMD GWAS and identify determinants of bone strength
Source: Nat Commun. 2021 Jun 7;12:3408. doi: 10.1038/s41467-021-23649-0 (PMC8184749; doi:10.1038/s41467-021-23649-0)
Supplement: Supplementary file 5 — Reporting Summary [file 41467_2021_23649_MOESM5_ESM.pdf]

nature research

Corresponding author(s): Charles R. Farber
Last updated by author(s): Apr 12, 2021

Reporting Summary

Nature Research wishes to improve the reproducibility of the work that we publish. This form provides structure for consistency and transparency in reporting. For further information on Nature Research policies, see our Editorial Policies and the Editorial Policy Checklist.

Statistics

For all statistical analyses, confirm that the following items are present in the figure legend, table legend, main text, or Methods section.

n/a	Confirmed	
☐	☒	The exact sample size (*n*) for each experimental group/condition, given as a discrete number and unit of measurement
☐	☒	A statement on whether measurements were taken from distinct samples or whether the same sample was measured repeatedly
☐	☒	The statistical test(s) used AND whether they are one- or two-sided<br>*Only common tests should be described solely by name; describe more complex techniques in the Methods section.*
☐	☒	A description of all covariates tested
☐	☒	A description of any assumptions or corrections, such as tests of normality and adjustment for multiple comparisons
☐	☒	A full description of the statistical parameters including central tendency (e.g. means) or other basic estimates (e.g. regression coefficient) AND variation (e.g. standard deviation) or associated estimates of uncertainty (e.g. confidence intervals)
☐	☒	For null hypothesis testing, the test statistic (e.g. *F*, *t*, *r*) with confidence intervals, effect sizes, degrees of freedom and *P* value noted<br>*Give P values as exact values whenever suitable.*
☒	☐	For Bayesian analysis, information on the choice of priors and Markov chain Monte Carlo settings
☒	☐	For hierarchical and complex designs, identification of the appropriate level for tests and full reporting of outcomes
☐	☒	Estimates of effect sizes (e.g. Cohen's *d*, Pearson's *r*), indicating how they were calculated

Our web collection on statistics for biologists contains articles on many of the points above.

Software and code

Policy information about availability of computer code

Data collection	Custom code was used to collect data from publicly available sources. Code is available on our GitHub (https://github.com/basel-maher/DO_project/).
Data analysis	Data analysis was performed using custom scripts and published packages written in the R programming language, which uses published code packages. The following R packages were used: DESeq2(version 1.20.0), FactoMiner(version 2.4), apeglm (version 1.4.2), qtl2(version 0.20), Argyle (version 0.2.2), sva(version 3.30.0), WGCNA(version 1.68), bnlearn(version 4.5), which included an implementation of the Max-Min Hill Climbing algorithm, igraph(version 1.2.4.1), stats(version 3.5.1), coloc(version 3.2.1), topGO (version 2.32.0), PhenStat(version 2.18.1), GenomicRanges(version 1.32.7), car (version 3.0.7), emmeans(version 1.4.1), and Seurat(version 3.1.4)<br><br>Furthermore, some analyses were performed using publicly available software packages written in various programming languages : FASTQC (version 0.11.5), MultiQC (version 1.0.dev0), HISAT2 (version 2.0.5), Stringtie (version 1.3.3), PEER (version 1.3), CellRanger(version 2.0.1), and STAR(version 2.5.1b)<br><br>Finally, we also used online tools: the UCSC liftOver tool (https://genome.ucsc.edu/cgi-bin/hgLiftOver) and ENSEMBLs Variant Effect Predictor tool (https://useast.ensembl.org/Tools/VEP).<br><br>Code is available on out GitHub (https://github.com/basel-maher/DO_project/).

For manuscripts utilizing custom algorithms or software that are central to the research but not yet described in published literature, software must be made available to editors and reviewers. We strongly encourage code deposition in a community repository (e.g. GitHub). See the Nature Research guidelines for submitting code & software for further information.

Data

Policy information about availability of data

All manuscripts must include a data availability statement. This statement should provide the following information, where applicable:

- Accession codes, unique identifiers, or web links for publicly available datasets
- A list of figures that have associated raw data
- A description of any restrictions on data availability

Raw genotyping data, calculated genotype and allele probabilities, and R/qtl2 cross files are available from Zenodo at DOI:10.5281/zenodo.4265417 [https://zenodo.org/record/4265417]. Raw sequencing data is available from the NCBI Gene Expression Omnibus database with accession codes GSE152708 [https://www.ncbi.nlm.nih.gov/geo/query/acc.cgi?acc=GSE152708] and GSE152806 [https://www.ncbi.nlm.nih.gov/geo/query/acc.cgi?acc=GSE152806] . Mapped DO mouse QTL and eQTL can be viewed at http://qtlviewer.uvadcos.io/.

eBMD GWAS summary statistics used for this study are available from GEFOS [http://www.gefos.org/?q=content/data-release-2018], as are the FN and LS BMD GWAS summary statistics [http://www.gefos.org/?q=content/data-release-2012].

We used bioGPS expression data from GEO with the accession code of GSE10246[https://www.ncbi.nlm.nih.gov/geo/query/acc.cgi?acc=GSE10246] to assay the expression of Sertad4, Glt8d2, and Qsox1 in osteoblasts and osteoclasts. We also downloaded the data from GEO with the accession code GSE54461[https://www.ncbi.nlm.nih.gov/geo/query/acc.cgi?acc=GSE54461] to query expression in primary calvarial osteoblasts. Glt8d2 knockout data was downloaded from the IMPC [https://www.mousephenotype.org/data/charts?accession=MGI:1922032&allele_accession_id=MGI:4364018&pipeline_stable_id=MGP_001&procedure_stable_id=IMPC_DXA_001&parameter_stable_id=IMPC_DXA_004_001&zygosity=homozygote&phenotyping_center=WTSI]. Mouse-Human homologs were obtained from MGI [http://www.informatics.jax.org/downloads/reports/HOM_MouseHumanSequence.rpt]. We also obtained data from the MGI Human-Mouse:Disease Connection database [http://www.informatics.jax.org/diseasePortal]. Gene Ontologies were obtained from AmiGO2 [http://amigo.geneontology.org/amigo].

Finally, we obtained expression data from version 7 of the Genotype-Tissue Expression project [https://gtexportal.org/home/datasets].

All figures are associated with analyzed data, not raw data.

Field-specific reporting

Please select the one below that is the best fit for your research. If you are not sure, read the appropriate sections before making your selection.

- [x] Life sciences
- [ ] Behavioural & social sciences
- [ ] Ecological, evolutionary & environmental sciences

For a reference copy of the document with all sections, see nature.com/documents/nr-reporting-summary-flat.pdf

Life sciences study design

All studies must disclose on these points even when the disclosure is negative.

Sample size	Power analyses performed a priori indicated that the sample size used (N~600) would provide adequate power to detect QTL with large to moderate effects on complex skeletal traits.<br><br>Sample sizes for each Qsox1 mutation were determined based on expected differences in ML. For microCT measurements, sample sizes were based on expected differences in the phenotypes.<br><br>For scRNA-seq, we estimated the number of cells to sequence based on how infrequent the rarest expected cell types were, based on prior work in the area. For RNA-seq, we chose to sequence a number of samples based on expected effect sizes for eQTL, based on the literature.
Data exclusions	In QTL mapping, due to the mapping algorithm, samples with missing weights were automatically excluded, due to use of weight as a covariate in the analysis. Sample sizes used for QTL mapping are available in Supplemental Table 1. The same exclusion criteria applied for the analysis of Qsox1 mutant mice.
Replication	In the context of QTL mapping, we did not replicate (generate a separate cohort to confirm associations) most associations. However, we had previously measured medial-lateral femoral width in an independent cohort of Diversity Outbred mice and a QTL scan of those data uncovered the presence of a similar QTL on Chr1. The identification of this locus across two different DO cohorts (which differed in generations, diets, and ages) provided robust replication justifying further analysis. Further discussion of this is in the main results and the Methods section.<br>RNA-seq included 192 replicates (96/sex), and scRNA-seq included 5 replicates. Qsox1 ML measurements included 530 replicates, while the microCT measurements included 50 replicates (25/genotype).<br>All replications were successful.

Randomization	The genotype of DO mice is inherently random. As a result, genotype was always randomized with respect to potential confounders (time of data collection, cage, data collection batch, etc.). For the characterization of Qsox1 knockout mice, we generated experimental mice by mating heterozygous mutants. As a result, Qsox1 mutant genotype was randomized across litter. The entire cohort of Qsox1 experimental mice where characterized for medial-later femoral width. For the analysis of microCT traits, we randomly chose an equal number of male mutant and wild-type mice (N=25/genotype).<br><br>For scRNA-seq, we randomly chose 5 DO mice. For RNA-seq experiments, we randomly chose 192 mice from the available set of mice at the time (number 1-417), with the constraint of having an equal number of males and females.
Blinding	All experiments used blinding. Blinding is inherently built into the design of the analysis of DO mice because the individuals collecting data were always blinded to the genotype of the animal. The same was done for the analysis of Qsox1 mutant mice, the individual collecting data was always blinded to Qsox1 genotype.

Reporting for specific materials, systems and methods

We require information from authors about some types of materials, experimental systems and methods used in many studies. Here, indicate whether each material, system or method listed is relevant to your study. If you are not sure if a list item applies to your research, read the appropriate section before selecting a response.

Materials & experimental systems

n/a	Involved in the study
☒	☐ Antibodies
☒	☐ Eukaryotic cell lines
☒	☐ Palaeontology and archaeology
☐	☒ Animals and other organisms
☒	☐ Human research participants
☒	☐ Clinical data
☒	☐ Dual use research of concern

Methods

n/a	Involved in the study
☒	☐ ChIP-seq
☒	☐ Flow cytometry
☒	☐ MRI-based neuroimaging

Animals and other organisms

Policy information about studies involving animals; ARRIVE guidelines recommended for reporting animal research

Laboratory animals	The study used 619 (315 males, 304 females) Diversity Outbred mice (Mus Musculus) (J:DO, JAX stock #0039376). Mice were sacrificed at approximately 12 weeks of age. Exact ages, weights, and other covariates are available in an RData file from http://qtlviewer.uvadcos.io. In the analysis of Qsox1 mutant mice, we collected data on 530 12 week old (median age = 12 weeks, mean age = 12.05 weeks) experimental mice (N males = 276, N females = 254).<br><br>We also used C57BL6/SJL mice (B6SJLF1/J, JAX Stock #100012). We only used F2 embryos that were obtained from the mating of B6SJLF1 females to B6SJLF1 males, in the generation of the aforementioned Qsox1 mutant mice.<br><br>All mice were maintained on a 12-hour light/12-hour dark cycle, at a temperature range of 60ºC-76ºC, with a humidity range of 20%-70%.
Wild animals	The study did not involve wild animals.
Field-collected samples	The study did not involve samples collected from the field.
Ethics oversight	The animal protocol for the characterization of Diversity Outbred mice and the generation and characterization of Qsox1 mutant mice was approved by the Institutional Animal Care and Use Committee (IACUC) at the University of Virginia

Note that full information on the approval of the study protocol must also be provided in the manuscript.
